# The Effects of Sodium Ions on Ligand Binding and Conformational States of G Protein-Coupled Receptors—Insights from Mass Spectrometry

**DOI:** 10.1021/jacs.0c11837

**Published:** 2021-03-12

**Authors:** Mark T. Agasid, Lars Sørensen, Leonhard H. Urner, Jun Yan, Carol V. Robinson

**Affiliations:** † Department of Chemistry, University of Oxford, 12 Mansfield Road, Oxford OX1 3TA, U.K.; ‡ Global Research Technologies, Novo Nordisk A/S, Novo Nordisk Park, Måløv 2760, Denmark

## Abstract

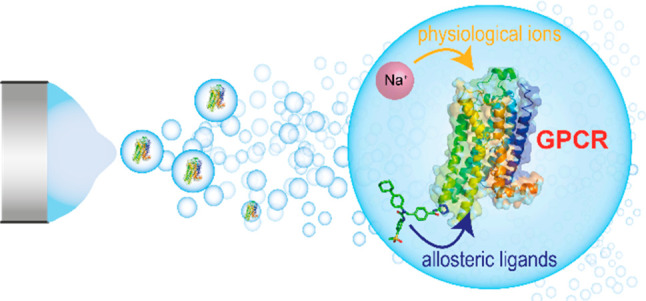

The use of mass spectrometry to investigate proteins is now well established and provides invaluable information for both soluble and membrane protein assemblies. Maintaining transient noncovalent interactions under physiological conditions, however, remains challenging. Here, using nanoscale electrospray ionization emitters, we establish conditions that enable mass spectrometry of two G protein-coupled receptors (GPCR) from buffers containing high concentrations of sodium ions. For the Class A GPCR, the adenosine 2A receptor, we observe ligand-induced changes to sodium binding of the receptor at the level of individual sodium ions. We find that antagonists promote sodium binding while agonists attenuate sodium binding. These findings are in line with high-resolution X-ray crystallography wherein only inactive conformations retain sodium ions in allosteric binding pockets. For the glucagon receptor (a Class B GPCR) we observed enhanced ligand binding in electrospray buffers containing high concentrations of sodium, as opposed to ammonium acetate buffers. A combination of native and -omics mass spectrometry revealed the presence of a lipophilic negative allosteric modulator. These experiments highlight the advantages of implementing native mass spectrometry, from electrospray buffers containing high concentrations of physiologically relevant salts, to inform on allosteric ions or ligands with the potential to define their roles on GPCR function.

More than 800 G protein-coupled receptors (GPCRs), encoded in the human proteome, regulate many physiological processes, making GPCRs intensively studied drug targets to combat human pathophysiology. Collectively, GPCRs account for 34% of all small-molecule drug targets.^1^ Beyond a traditional orthosteric ligand binding site, GPCRs also harbor allosteric pockets that bind a host of endogenous ions, peptides, lipids, and intracellular proteins that coregulate GPCR function.^2^ One of the most well-known, but enigmatic examples involves a conserved sodium binding pocket in Class A GPCRs. High-resolution X-ray crystallography structures of several GPCRs identified this highly conserved pocket that is occupied by a Na^+^ ion in the inactive conformation.^3^ In active state conformations, this sodium ion-binding pocket collapses, with possible egress of the ion into the cytoplasm.^4^ This binding pocket is important with functional and mutagenesis studies revealing the inverse agonist effects of sodium ions on GPCRs. Its functional importance has been described as an ionic microswitch that couples the extracellular domain with the intracellular structural changes associated with heterotrimeric G protein coupling.^5,6^ However, it has been difficult to affirm concurrent changes in receptor sodium occupancy by traditional biochemical or functional assays.^3^ Moreover since other allosteric binding sites have been reported for divalent cations, and lipophilic positive and negative allosteric modulators,^7−10^ controlling these allosteric sites to modulate GPCR function serves as a potential route for therapeutic intervention.^11^

Native mass spectrometry (nMS) has become a powerful biophysical tool to characterize membrane proteins, including relatively few examples of GPCRs.^12,13^ nMS exploits the gentle ionization conditions enabled by nanoelectrospray ionization (nESI),^14^ which can preserve noncovalent interactions, informing on protein–ligand and protein–protein interactions.^15^ The high resolution afforded by state-of-the-art instruments have recently identified a previously unknown GPCR-lipid interaction that enhances coupling to Gα_s_,^16^ and defined the effect of post-translational modifications on ligand binding to GPCRs.^17^ Very recently, hybridization of nMS with “omics” based platforms has enabled tandem MS (MS^n^) to perform nMS, proteoform and ligand identification.^18^

In general, nMS experiments rely on exchanging proteins from biochemical assay buffers (i.e., >100 mM salt) into MS-compatible solutions composed of volatile agents, e.g., ammonium acetate (NH_4_OAc), to avoid detrimental effects of nonvolatile salts during the nESI process.^19^ These adverse effects include generation of salt clusters and the formation of nonspecific adducts with protein ions, which distribute the signal over multiple adduct states, thereby suppressing ion intensity, decreasing mass accuracy, resolution, and detection limits. To overcome these experimental limitations, nanoscale nESI tips (60–600 nm) (nanoemitters) have shown initial promise for their greater salt tolerance, owing to the smaller electrospray droplets generated.^20−22^

Here we describe the use of ∼100 nm diameter nanoemitters to study two GPCRs—a thermostabilized variant of the prototypical Class A GPCR, the adenosine 2A receptor (A2aR),^23^ and a Class B GPCR, the wild-type glucagon receptor (GCGR). We have exploited the benefits of the nanoemitters to show how ligand type affects sodium occupancy of A2aR and how physiologically relevant solution conditions preserve lipophilic drug binding to GCGR.

Since high-resolution structures of A2aR, captured in inactive and active conformations, were the first to underscore the importance of sodium ions in modulating function, we used this receptor for our initial investigation (Figure 1A). We first optimized the nanoemitter orifice size and compared results with spectra recorded for microemitter tips in NH_4_OAc on a Q-Exactive UHMR mass spectrometer (Figure S1). Nanoemitter diameters of 115 ± 11 nm yielded reproducible spectra for most soluble and membrane proteins examined, including A2aR (Figure S2). Importantly nanoemitters produced similar mass A2aR (46526.7 ± 0.5 Da vs 46527.1 ± 0.5 Da) and average charge states (*Z*_ave_ 12.33 ± 0.13 vs 12.41 ± 0.16). The extent of collisional activation required to remove LMNG detergent from the A2aR was also similar (Figure S3 and S4). Remarkably, A2aR electrosprayed from NaCl/Tris solutions produced an apo state protein ion (i.e., no sodium ions bound) as well as protein ions with up to seven readily resolved sodium binding events (Figure S5). Additionally, lipid adducts that copurified with A2aR were readily discerned in mass spectra (Figure S4). These results illustrate the capability of nanoemitters to produce high-resolution spectra directly from high concentrations of sodium that better mimic biochemical assay conditions.^19,21^

**Figure 1:**
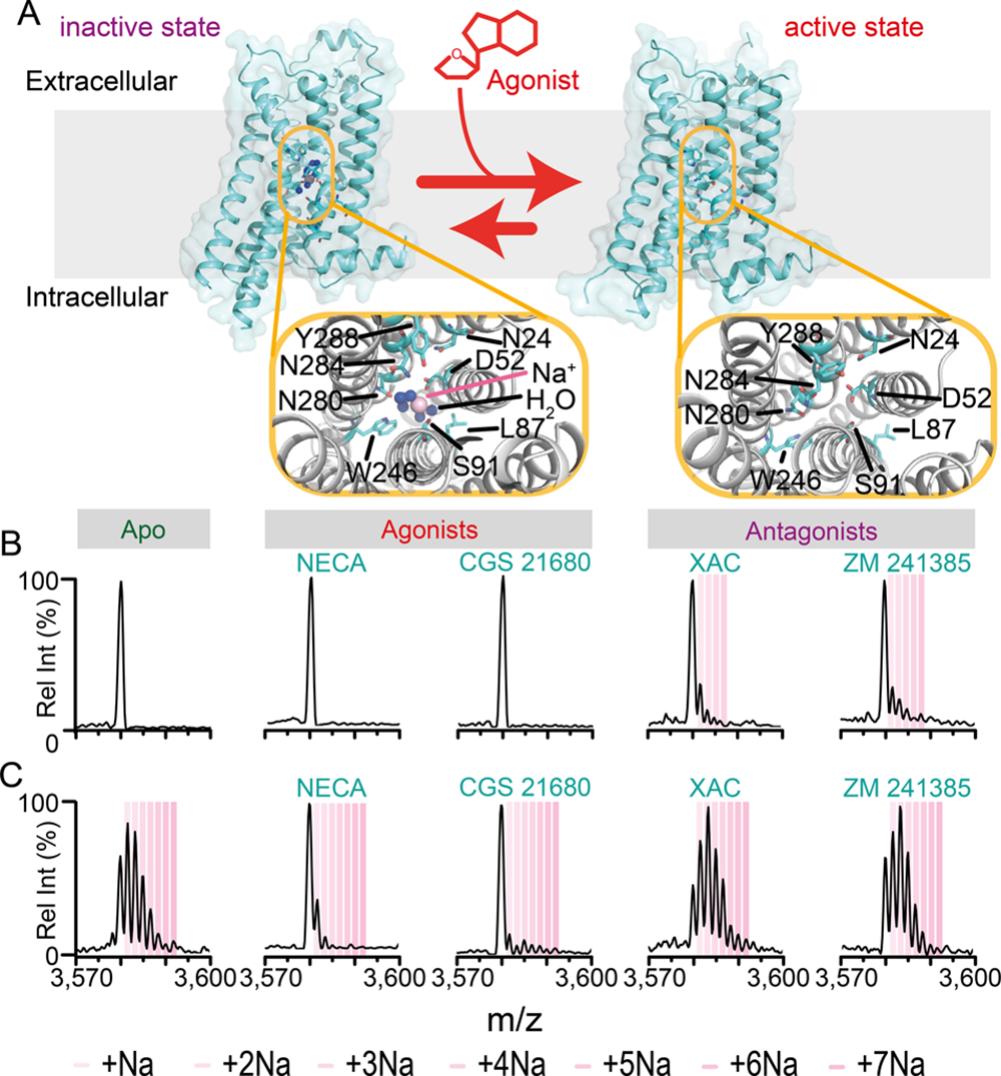
Ligand dependent effects on the sodium bound states of A2aR. (A) Schematic illustrating the sodium binding pocket of A2aR in an inactive conformation (PDB 4EIY) and the collapse of the pocket upon adopting an active conformation (PDB 3QAK). 13+ charge state of A2aR electrosprayed from (B) 200 mM NH_4_OAc and (C) 50 mM NaCl, 5 mM Tris (both pH 7.5 with 2× CMC LMNG) in the absence or presence of 10 μM ligand. Spectra are representative of *n* ≥ 4 nanoemitters and 2 protein preparations.

We next investigated differences in sodium binding properties induced by agonists and antagonists (structures Figure S6). A2aR incubated with 10 μM of the nonselective agonist NECA (*K*_d_ ≈ 100 nM)^24^ or the highly selective A2aR agonist CGS 21680 (*K*_d_ ≈ 65–117 nM)^25^ produced spectra from NH_4_OAc solutions devoid of any bound sodium ions, similar to A2aR without ligand (Figure 1B). Interestingly, incubation with the antagonists XAC (*K*_d_ ≈ 10 nM)^24^ or ZM241385 (*K*_d_ ≈ 2 nM)^24^ retained at least one sodium on the receptor, even after buffer exchange into NH_4_OAc. Spectra obtained in NaCl/Tris with antagonists consistently showed a much greater retention of sodiums (from 1 to 7), whereas the presence of agonist significantly attenuated the intensities of sodium bound states, especially for CGS 21680 (Figure 1C and Figures S7–S10). The sodium adduct intensities were minimally affected by increased collisional activation (Figure S11). Intriguingly these results provide complementary evidence for changes in the sodium-bound states between active and inactive Class A GPCRs.

While nMS aims to retain noncovalent interactions some interactions have been challenging to preserve, particularly small-molecule ligands bound to membrane proteins in detergent micelles. Although some exceptions exist,^17^ in these experiments we did not detect binding of the agonists and antagonists (Figure S4C,D), yet we observed clear effects of their presence on sodium binding. Previous studies on membrane protein–lipid binding events highlighted the complex interplay of lipid headgroup chemistry, available charged residues at the protein surface, and electrospray polarity (i.e., positive or negative).^26^ As the structures of A2aR ligand employed in this study (Figure S6) have a propensity to be positively charged, this may inhibit binding to postiviely charged protein ions explaining the absence of peaks corresponding to ligand binding.

To confirm that the differences observed in A2aR sodium occupancy resulted from ligand-induced conformational changes we performed a competition assay. We incubated the receptor in NH_4_OAc first with antagonist ZM241385 and then challenged the solution with 40-fold excess of the agonist NECA. Comparing the sodium binding peaks following incubation with ZM241385 alone and after challenge with NECA (Figures S12) reveals depletion of sodium binding. This reduction is consistent with solution phase experiments depicting collapse of allosteric binding pocket and egress of the sodium ion when A2aR adopts an active conformation in the presence of high concentrations of agonist, in anticipation of G protein coupling.^5^ In NaCl/Tris solutions, the loss of more than one sodium ion between agonized and antagonized states is consistent with the NH_4_OAc experiments (Figure 1C). Moreover, the similarity in sodium adduct pattern in the apo (ligand-free state) versus antagonized states for NaCl/Tris, reflects the inverse agonist pharmacology of sodium ions that stabilize an inactive conformation.^27^ Furthermore, molecular dynamics (MD) simulations along the conformational landscape of A2aR indicate the potential for more than one sodium ion-binding site beyond the canonical site identified by X-ray crystallography.^28^

Turning to the glucagon receptor (GCGR), we optimized solution conditions and found that the recently developed oligoglycerol detergent, G1,^29^ and cholesteryl hemisuccinate (CHS) mixed micelle composition enabled us to liberate GCGR into the gas-phase from both NH_4_OAc and NaCl/Tris (Figure 2B,C). To test receptor functionality we confirmed binding of the endogenous peptide, glucagon (Figure S13). Unlike A2aR we did not observe sodium binding to GCGR, allowing direct comparison of spectra from both solution conditions. The measured mass of the receptor was 52415.4 ± 0.5 Da and 52414.9 ± 0.3 Da from both electrospray solutions. Additionally, at equivalent collisional activation conditions, GCGR ions produced from NH_4_OAc exhibited a bimodal charge state distribution with higher charge states observed than NaCl/Tris (22+ to 13+ vs 19+ to 11+). This bimodal distribution likely indicates an unfolded receptor population. In NH_4_OAc and NaCl/Tris, a series of equally spaced peaks, ∼203 Da apart, was apparent (Figure 2B,C insets), and assigned to *N*-acetyl-d-glucosamines (HexNac) at four known glycosylated sites on the extracellular domain.^30^ Interestingly, for spectra recorded in NaCl/Tris, a second adduct series at 579.1 ± 1.7 Da, with up to two binding events, was apparent for charge states ≤16+. Collisional activation revealed that the unknown molecule could be dissociated, suggesting that it was noncovalently bound to the protein (Figure S14A,B). On the basis of the mass, we suspected this molecule was the negative allosteric modulator, NNC0666 (monoisotopic mass = 579.22 Da), present in purification solutions to stabilize GCGR. NNC0666 is a variant of the lipophilic compound NNC0640 (Figure 2A), which is an essential buffer supplement for stabilizing apo GCGR during purification and crystallization processes.^31,32^

**Figure 2:**
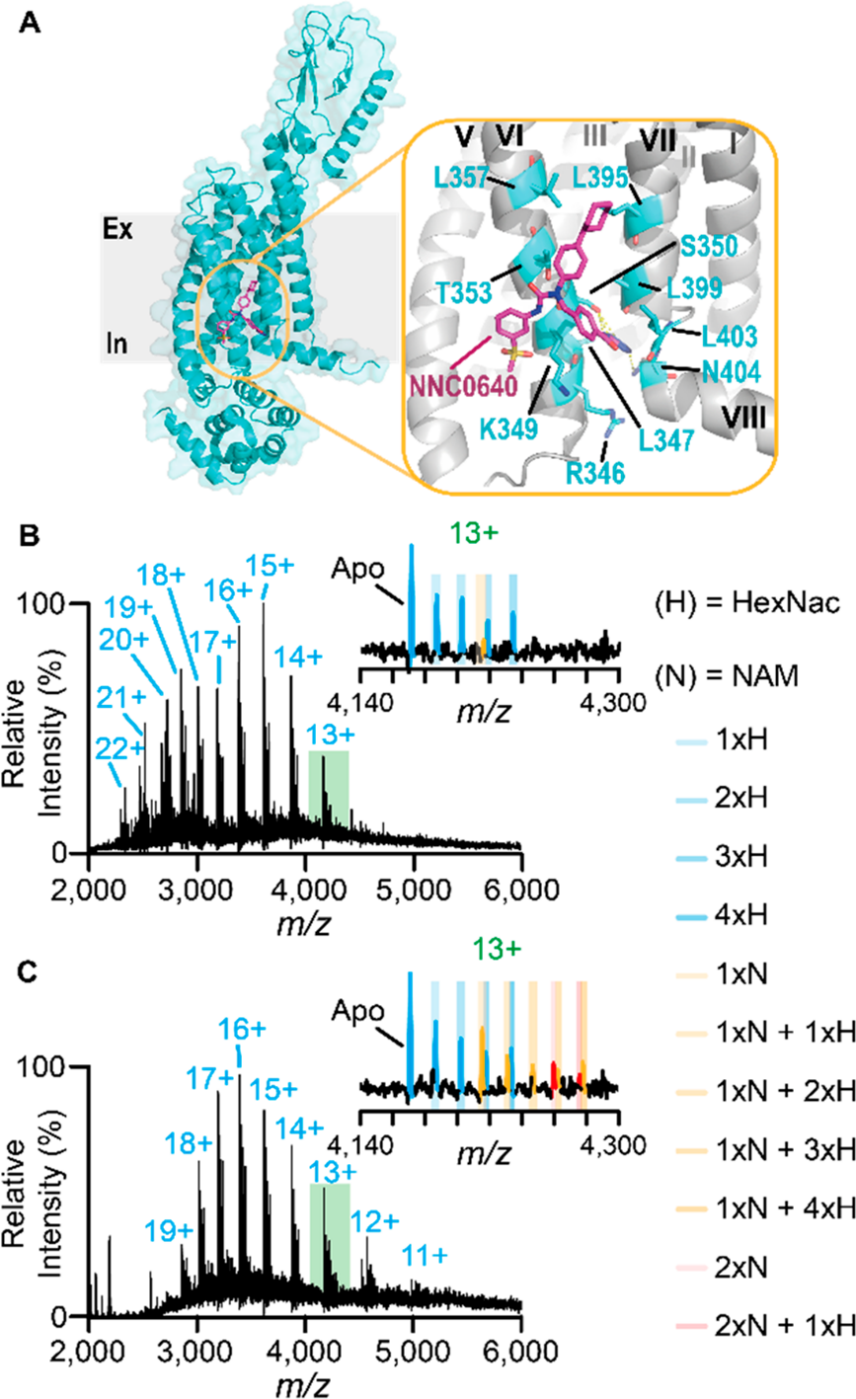
Preservation of lipophilic drug binding to the glucagon receptor in NaCl-containing electrospray buffers. (A) Structure of the full-length glucagon receptor (PDB 5XEZ) showing the allosteric binding pocket binding NNC0640, a variant of to NNC0666, situated between transmembrane loops VI and VII with interacting residues highlighted in blue. Native mass spectra of GCGR released from G1/CHS micelles in (B) 250 mM NH_4_OAc, pH 7.5 electrospray buffer or (C) 50 mM NaCl, 5 mM Tris, pH 7.5 electrospray buffers with insets expanding the 13+ charge state. Differences in binding of the NNC066 are more prevalent in (C), highlighted orange and red for one and two NAMs, respectively. Spectra are representative of *n* ≥ 4 nanoemitters and *n* = 2 protein preparations.

To identify ligands associated with GCGR we performed a tandem MS experiment on an Orbitrap Eclipse Tribrid MS platform.^18^ Broad isolation of the 13+ charge state (selection window *m*/*z* = 100–200) released many species below *m*/*z* = 1000 (Figure S15A,B). Focusing on the *m*/*z* 574–584 region fragmentation suggested potential lipid fragmentation patterns (Figure S16), whereas fragmentation of *m*/*z* 580 ion yielded a pattern consistent with NNC0666 (Figure S15C–E), an assignment subsequently corroborated using a standard solution of NNC0666 (Figure S15F–J).

To compare directly how solution conditions affect binding we quantified the fractional binding of NNC0666 to GCGR in the two different solutions (Figure S17C). We found that 1 μM NNC0666 in NaCl/Tris or NH_4_OAc solution achieved 40 ± 5% and 8 ± 2% fractional binding at 1 μM, respectively, with an expected value of 17% based on the *K*_d_ value (3–10 nM). Thus, we observe an approximately 5-fold enhancement in the presence of sodium ions.

For Class B GPCRs, sodiums are not expected to affect binding affinity, as in documented cases of monovalent and divalent cations with other GPCRs.^33,34^ Thus, it is intriguing that switching from NH_4_OAc to NaCl/Tris affects ligand binding with GCGR. Reduction of the GCGR charge state distribution in NaCl/Tris electrospray buffers suggests that the gas-phase ions produced are more compact. Supplementing higher concentrations of NNC0666 to NH_4_OAc buffers appears to have a similar effect on the charge state distribution (Figure S17A), possibly through better stabilization of the receptor fold during the nano-ESI process. Hence, these results imply that the structural properties necessary for preserving ligand binding in dynamic proteins, such as GPCRs, are better maintained in NaCl/Tris than NH_4_OAc buffer.

Overall, these GPCR examples demonstrate that a fundamental challenge for MS, the use of high salt, can be ameliorated through the use of nanoemitters.^19^ Importantly, we also demonstrate with A2aR that nMS can complement other biophysical tools to inform on allosteric effects at the level of individual sodium ions. In the case of GCGR, we show improved lipophilic ligand binding in the sodium-containing buffer, which has implications for preserving protein–ligand binding interactions in drug discovery or the characterization of endogenous ligands from biological systems. We envisage that these developments will help delineate the complex interplay between physiological ions and ligands on modulating GPCRs, and more generally on membrane protein structure and function of other receptors, transporters, and ion channels.
