# Supplementary material for: The Effects of Sodium Ions on Ligand Binding and Conformational States of G Protein-Coupled Receptors—Insights from Mass Spectrometry
Source: J Am Chem Soc. 2021 Mar 12;143(11):4085–9. doi: 10.1021/jacs.0c11837 (PMC7995251; doi:10.1021/jacs.0c11837)
Supplement: Supplementary file 1 — ja0c11837_si_001.pdf [file ja0c11837_si_001.pdf]

Supporting Information

for

The effects of sodium ions on ligand binding and conformational states of GPCRs – insights from mass spectrometry

Mark T. Agasid,[†] Lars Sørensen,[§] Leonhard H. Urner,[†] Jun Yan,[§] Carol V. Robinson[*,†]

[†] Department of Chemistry, University of Oxford, 12 Mansfield Road, Oxford OX1 3TA, U.K.

[§]Global Research Technologies, Novo Nordisk A/S, Novo Nordisk Park, Måløv 2760, Denmark.

Table of Contents

Materials and Methods	**Page**
Supplementary Figure 1. Spectra variability for A2aR electrosprayed from ca. 1 µm microemitter tips	3 – 7
Supplementary Figure 2. SEM micrograph of nanoemitter tips	8
Supplementary Figure 3. Native MS spectra of A2aR	
Supplementary Figure 4. Native MS spectra of A2aR incubated with ligands	9
Supplementary Figure 5. Spectra variability for A2aR electrosprayed from	10
nanoemitter tips	11
Supplementary Figure 6. Chemical structures of A2aR ligands	12
Supplementary Figure 7. Spectra variability for A2aR electrosprayed from nanoemitter tips in the presence of 10 µM NECA	13
Supplementary Figure 8. Spectra variability for A2aR electrosprayed from nanoemitter tips in the presence of 10 µM CGS21680	14
Supplementary Figure 9. Spectra variability for A2aR electrosprayed from nanoemitter tips in the presence of 10 µM XAC.	15
Supplementary Figure 10. Spectra variability for A2aR electrosprayed from nanoemitter tips in the presence of 10 µM ZM241385	16
Supplementary Figure 11. Increased collisional activation minimally affects sodium adducts on A2aR	17
Supplementary Figure 12. ZM241385/NECA competitive inhibition assay in NH_4OAc electrospray buffer showing tip to tip variability	18
Supplementary Figure 13. Binding of glucagon to the glucagon receptor	19
Supplementary Figure 14. Collisional activation energy ramp and charge state dependence of negative allosteric modulator (NAM) binding to GCGR	20
Supplementary Figure 15. Dissociation and fragmentation of ligands from GCGR using a Thermo Eclipse Orbitrap Tribrid mass spectrometer	21
Supplementary Figure 16. Fragmentation patterns of representative species in the m/z 574 -579 range dissociated from GCGR highlighted in the	22
Supplementary Figure 15	23
Supplementary Figure 17. Native mass spectrum of GCGR incubated with 10 µM NNC0666 in NH_4OAc	
Supplementary Figure 18. Proposed fragments of NNC0666	24
References	
	25
	26

Materials and Methods

Materials

All reagents were purchased from Sigma Aldrich and used as received without any further processing, unless specified.

Sodium chloride, potassium chloride, magnesium chloride, (4-(2-hydroxyethyl)-1-piperazineethanesulfonic acid) (HEPES), ethylenediaminetetraacetic acid (EDTA), tris(hydroxymethyl)aminomethane (Tris), ammonium acetate (7.5 M), imidazole, dimethylsulfoxide (DMSO), glucagon (1-29, synthetic peptide), carbonic anhydrase (bovine), concanavalin A (jack bean), alcohol dehydrogenase (yeast), chaperonin 60 (GroEL), and platinum wire (purity 99.9%) were purchased from Sigma Alrich (St Louis, MO). The detergents lauryl maltose neopentyl glycol (LMNG), n-dodecyl-β-D-maltoside (DDM), and cholesteryl hemisuccinate tris salt (CHS) was purchased from Anatrace (Maumee, OH). EDTA-free protease inhibitor tablets were purchased from Roche. Adenosine 2A ligands NECA, CGS 21680, and ZM241385 were purchased from Tocris Biosciences (Abingdon, UK). The A2aR antagonist XAC was purchased from Insight Biotechnology (Wembley, UK). Glass capillary for fabricating nanoelectrospray emitters was purchased from Harvard Apparatus (Cambridge, MA). Amicon Ultra molecular weight cutoff filters were purchased from Merck. Zeba desalting columns (7.5 kDa cut off, 75 μL) were purchased from Thermo Scientific. Disposable polypropylene columns (10 mL) were purchased from Biorad (Kidlington, UK). Ni-NTA immobilized metal affinity chromatography resin was purchased from Qiagen (Manchester, UK). Negative allosteric modulator, NNC0666, was a kind gift from Novo Nordisk.

Expression and purification of the adenosine 2A receptor

A thioredoxin-A2aR fusion protein bearing a N-terminal cleavable leader sequence gp67 followed by a 10xHis tag and TEV protease cleavage site, a thioredoxin and wild-type human A2aR (residues 6-316) through an EAAAKA linker was prepared, as previously described. A potential N-linked glycosylation site was also mutated (N154A).[1] The construct was ligated into the pFastBac1 expression vector and protein expression used the Bac-to-Bac baculovirus expression system and Sf9 cells. Cells were harvested by 60 hours post infection by centrifugation at 3,000 xg for 20 min at 4 ^{o}C. The cell pellet was resuspended in hypotonic buffer (20 mM HEPES, 1 mM EDTA, 1 mM PMSF, pH 7.5), flash-frozen, and stored at -80 ^{o}C until further use.

Cells were lysed by dounce homogenization, debris removed by centrifugation at 3,000 xg for 30 min at 4 ^{o}C, and membranes pelleted by ultracentrifugation at 100,000 xg for 1 h at 4 ^{o}C. Membranes were resuspended in a high-salt buffer (500 mM NaCl, 25 mM Tris, 10 mM KCl and 5 mM $MgCl_2$ pH 7.4) and then pelleted a second time. Membranes were resuspended in buffer

(300 mM NaCl, 20 mM HEPES, 1 mM PMSF, 100 µM NECA, an EDTA-Free cOmplete protease inhibitor tablet) flash-frozen and stored at -80 °C until further use.

For purification, membranes were thawed and solubilized by 1% LMNG with gentle agitation for 3 h at 4 °C. Insoluble material was removed by centrifugation at 20,000 xg for 20 min at 4 °C. The supernatant was collected, filtered through a 0.46 µm dual-stage filter (Millipore), and then incubated with Ni-NTA resin for 4 h at 4 °C with gentle stirring. The resin was collected, packed into a disposable column, then washed with 10 column volumes (CV) with wash buffer (500 mM NaCl, 20 mM HEPES, 10% (v/v) glycerol, 80 mM imidazole, 100 µM NECA, 0.01% (w/v) LMNG, 40 mM imidazole, pH 7.5). The protein was eluted with elution buffer (100 mM NaCl, 20 mM HEPES, 10% (v/v) glycerol, 100 µM NECA, 250 mM imidazole, 0.01% (w/v) LMNG, pH 7.5). Protein was concentrated and imidazole removed using a PD10 column (GE Healthcare). The 10xHis tag was removed by overnight incubation with TEV protease using dialysis buffer (100 mM NaCl, 20 mM HEPES, 10% (v/v) glycerol, 100 µM NECA, 0.01% (w/v) LMNG, 0.2 mM TCEP). TEV protease was removed by reverse IMAC on Ni-NTA resin. The protein was concentrated and then loaded onto a Superdex G200 Increase column (GE Healthcare) equilibrated with SEC buffer (100 mM NaCl, 20 mM HEPES, 10% (v/v) glycerol, 0.01% (w/v) LMNG). Peak fractions were pooled, protein was concentrated, and aliquoted. Aliquots were flash frozen in the presence of 100 µM NECA.

Expression and purification of the glucagon receptor

The glucagon receptor construct was prepared using a N-terminal HA signal peptide followed by a HPC4 - affinity tag, 3C protease cleavage site, and wild-type human glucagon receptor (residues 26 – 477). The construct was ligated into the pFastBac1 vector and expressed using the Bac-to-Bac baculovirus system using Sf9 cells. Cells were collected 72 h post-infection by centrifugation at 4,600 x g at 4 °C, resuspended in PBS pH 7.4, pelleted again, and then the pellet was flash-frozen and finally stored at -80 °C until further use.

Membranes were prepared by dounce homogenization using two buffers. First the thawed cell pellet was dounced in low-salt buffer (25 mM Tris, 10 mM KCl and 5 mM $MgCl_2$ pH 7.4 supplemented with an EDTA-free cOmplete Protease Inhibitor Tablet), centrifuged at 20,000 xg and 4 °C, supernatant discarded, the pellet resuspended in high-salt buffer (500 mM NaCl, 25 mM TRIS, 10 mM KCl and 5 mM MgCl2 pH 7.4 supplemented with EDTA-free cOmplete protease inhibitor tablet) and dounced again. The high-salt buffer wash was repeated twice, then membranes were resuspended in low-salt buffer with 33% (v/v) glycerol before being flash frozen and stored at -80 °C until use.

For purification, the membranes were diluted with an equivolume solution of 2% (w/v) DDM, 0.04% (w/v) CHS, 150 mM NaCl, 25 mM Tris, pH 7.4 and gently stirred for 2 h at 4 °C. Insoluble debris was removed by centrifugation at 20,000 xg for 20 min at 4 °C and incubated with anti-HPC4 affinity resin (prepared at Novo Nordisk) overnight in the presence of 150 mM NaCl, 25

mM Tris, 10 mM $CaCl_2$, 10% (v/v) glycerol, 0.05% (w/v) DDM, 0.01% (w/v) CHS, and 1 μM NNC0666, pH 7.4. The following day, the resin was collected by centrifugation at 800 xg and 4 °C, the supernatant discarded, and the beads added to a disposable column. The resin was washed with 25 CVs of wash buffer (150 mM NaCl, 25 mM Tris, 10 mM $CaCl_2$, 10% (v/v) glycerol, 0.05% (w/v) DDM, 0.01% (w/v) CHS, and 1 μM NNC0666) before eluting with 5 CVs of elution buffer (150 mM NaCl, 25 mM Tris, 25 mM EDTA, 0.04% (w/v) G1,[2] 0.08% (w/v) CHS, 10% (v/v) glycerol, and 1 μM NNC0666). The protein was concentrated using a 100 kDa MWCO filter, then loaded onto a Superdex G-200 Increase 10/300 column, equilibrated with 150 mM NaCl, 25 mM Tris, 0.04% (w/v) G1, 0.08% (w/v) CHS, and 1 μM NNC0666. Peak fractions were stored in aliquots, flash-frozen, and stored at -80 °C until further use.

Sample preparation

Proteins were buffer exchanged into 200 – 250 mM ammonium acetate, pH 7.5, or 50 mM NaCl, 5 mM Tris, pH 7.5, with corresponding detergent buffers using Zeba desalting columns (Pierce, 7.5 kDa MWCO, 75 μL).

For A2aR studies, protein was exchanged into solutions containing 2x CMC LMNG. In ligand incubation studies, NECA, CGS 21680, XAC, and ZM241385 were prepared as DMSO stock solutions at >30 mM concentration. The ligand stock solutions were subsequently serially diluted to 100 μM solutions in 200 mM NH_4OAc, pH 7.5 or 50 mM NaCl, 5 mM Tris, pH 7.5 and 2x CMC LMNG. Receptor (2 – 5 μM) was incubated with 10 μM of each corresponding ligand for 30 min on ice before analyzing by native mass spectrometry. For competition assays, the receptor was incubated with ZM241385 and NECA for 1 h on ice before analyzing by native mass spectrometry.

For GCGR, protein was exchanged into either 250 mM NH_4OAc, pH 7.4 or 50 mM NaCl, 5 mM Tris, pH 7.5 supplemented with 0.04% (w/v) G1 and 0.08% (w/v) CHS and 1 μM NNC0666. Stock solutions of NNC0666 were prepared at 10 mM in DMSO. The DMSO stock was diluted in detergent-supplemented solutions of NH_4OAc or NaCl/Tris to 100 μM NNC0666. Receptor (4 - 6 μM) was infused into the mass spectrometer as is for 1 μM NNC0666. For higher concentrations of NNC0666, the receptor was first incubated with 10 μM NNC0666 for 1 h on ice before infusing into the mass spectrometer. For GCGR binding studies, glucagon peptide was dissolved in DMSO to a final concentration of 10 mM. Glucagon peptide was serially diluted in 250 mM NH_4OAc supplemented with G1/CHS. The peptide solution was then added directly at 4 °C to desalted GCGR (4 - 6 μM) resulting in a solution with the desired glucagon concentration, in 0.1% (v/v) DMSO, and incubated for 30 min prior to native MS.

Native mass spectrometry

Nanoelectrospray ionization tips were prepared in-house from 1.2 mm o.d. x 0.86 mm i.d. filamented capillary (Harvard Biosciences) using a P-2000 CO2 laser puller. Optimal nESI tip diameters for infusing proteins from high-salt electrospray solutions were empirically determined

using standard native mass spectrometry soluble proteins (carbonic anhydrase, concanavalin A, yeast alcohol dehydrogenase, and GroEL) using previously published descriptions as a starting point (data not shown).[3-5]

Samples were directly infused into a Q-Exactive UHMR hybrid quadraupole-Orbitrap mass spectrometer (Thermo). To initiate an electrospray, a 0.127 mm diameter Pt wire was placed in the capillary and a capillary voltage of 0.6 – 1.0 kV was applied for ~115 nm tips and 1.0 – 1.2 kV for ~1 μm tips. Ions were transferred to the higher-energy collisional dissociation (HCD) cell following a gentle voltage gradient (injection flatapole, inter-flatapole lens, bent flatapole, transfer multipole: 5, 3, 2, 1 V, respectively). To facilitate detergent micelle stripping, infused GPCRs were activated in the HCD cell using an acceleration voltage of 200 V for LMNG or a combination of in-source trapping (-100 V) and HCD activation energy (110 V) for G1/CHS mixed micelles. Transient times were 64 ms (resolution setting = 17,500 at *m/z* 200) with an AGC target of $1x10^5$ and fill time of 100 ms, unless otherwise specified. Spectra were acquired at 10 microscans, and averaged with a noise level parameter of 3 (reduced from default value of 4.64). Other instrument parameters include capillary temperature 100 ^{o}C, S-lens RF 200%, and UHV pressure 1.1×10^{-9} mbar.

Small-molecule identification for GCGR experiments was performed on an Eclipse Orbitrap Tribrid mass spectrometer (Thermo) as previously described.[6] The typical instrument settings were: transfer capillary temp 200 ^{o}C, RF lens value of 150%, 100 – 150 V source fragmentation to strip the detergent micelle along with Source Compensation setting of 0.15 to aid protein transfer, instrument set to Intact protein mode (IRM cell 20 mTorr), and trapping, isolation, and activation of proteins in ligands in the quadrupole-linear ion trap. To dissociate ligands, the ion trap mass range was set to 'high m/z,' the 13+ charge of GCGR (*m/z* 4140) with a *m/z* 100-200 selection window was chosen, collisional activation was performed in the IRM (HCD 25 – 30%) or ion-trap (CID 25 – 30), AGC targets of 100 – 300% and maximum inject times of 100 - 300 ms. Dissocated ligands were trapped in the ion-trap set to 'normal range' and 'enhanced' resolution and individual peaks were further isolated using a selection window of m/z = 1 for ligand fragmentation in pMS^4 and pMS^5.

Data Analysis

Native MS data was analysed using a combination of UniDec software and Origin Labs (v.2020).

For GCGR fractional binding studies, the 13+ charge state was chosen for analysis as this is the lowest charge state in NH_4OAc spectra that showed NNC0666 binding. The background was subtracted using UniDec and the peak heights were determined for each state of GCGR (i.e. Apo, 1x NAM, 2x NAM). The fractional binding was determined by reporting the fraction of each state divided by the total intensity of all observed states after background subtraction.

Spectral matching

To identify NNC0666 bound to GCGR, the psuedoMS[4] (pMS[4]) and pMS[5] spectra were compared with the fragmentation spectra of the neat compound. NNC0666 from a 10 mM stock in DMSO was diluted to 1 µM in 50:50 methanol: water and 0.1% TFA and directly infused into an Eclipse Tribrid Oribtrap mass spectrometer at 1.2 kV from a gold-coated capillary. The $[M+H]^+$ = 580 *m/z* ion was selected in the ion trap with an isolation width of *m/z* = 1, fragmented in the IRM cell at 30% HCD or ion trap at 25 -30 CID, and detected in the ion trap at normal *m/z* range and tuned to AGC (100%), maximum injection time (100 – 300 ms), and averaging of 100 scans, depending on signal intensity.

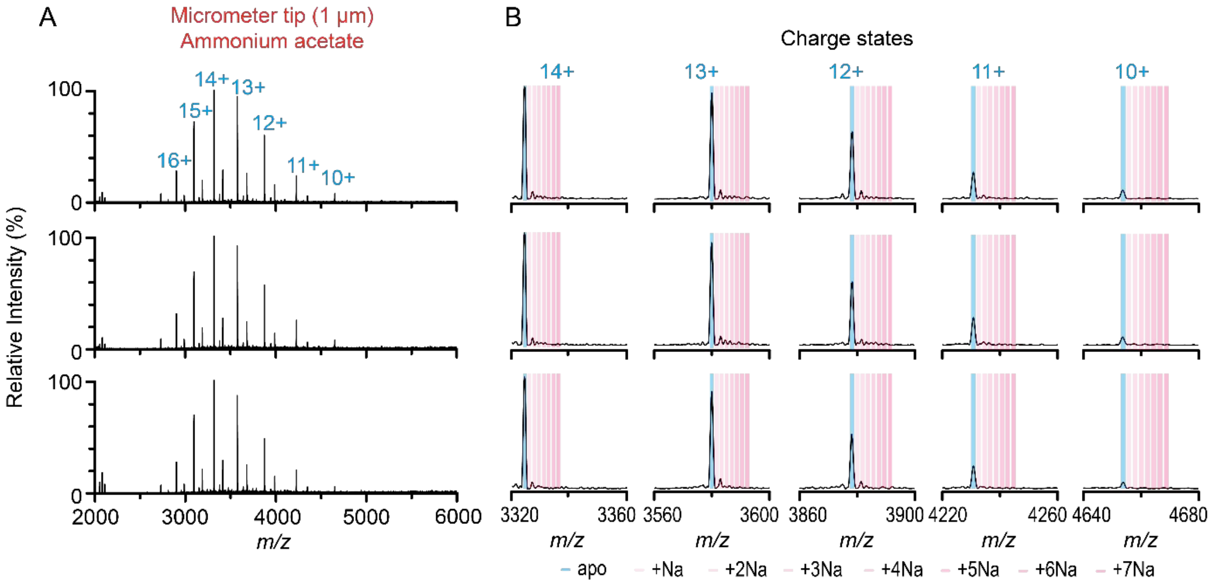

Supplementary Figure 1. Tip to tip variability for A2aR electrosprayed from ca. 1 µm microemitter tips following a desalting step. Three replicate experiments were performed for the same solution electrosprayed from three different tips illustrating the reproducibility and minimal carryover of sodium ions in the electrospray buffers. A small contaminant of 38 ± 1 Da may be found on the receptor, indicative of potassium. Spectra were recorded using the same native MS parameters and n > 4 microemitters and result from same protein preparation.

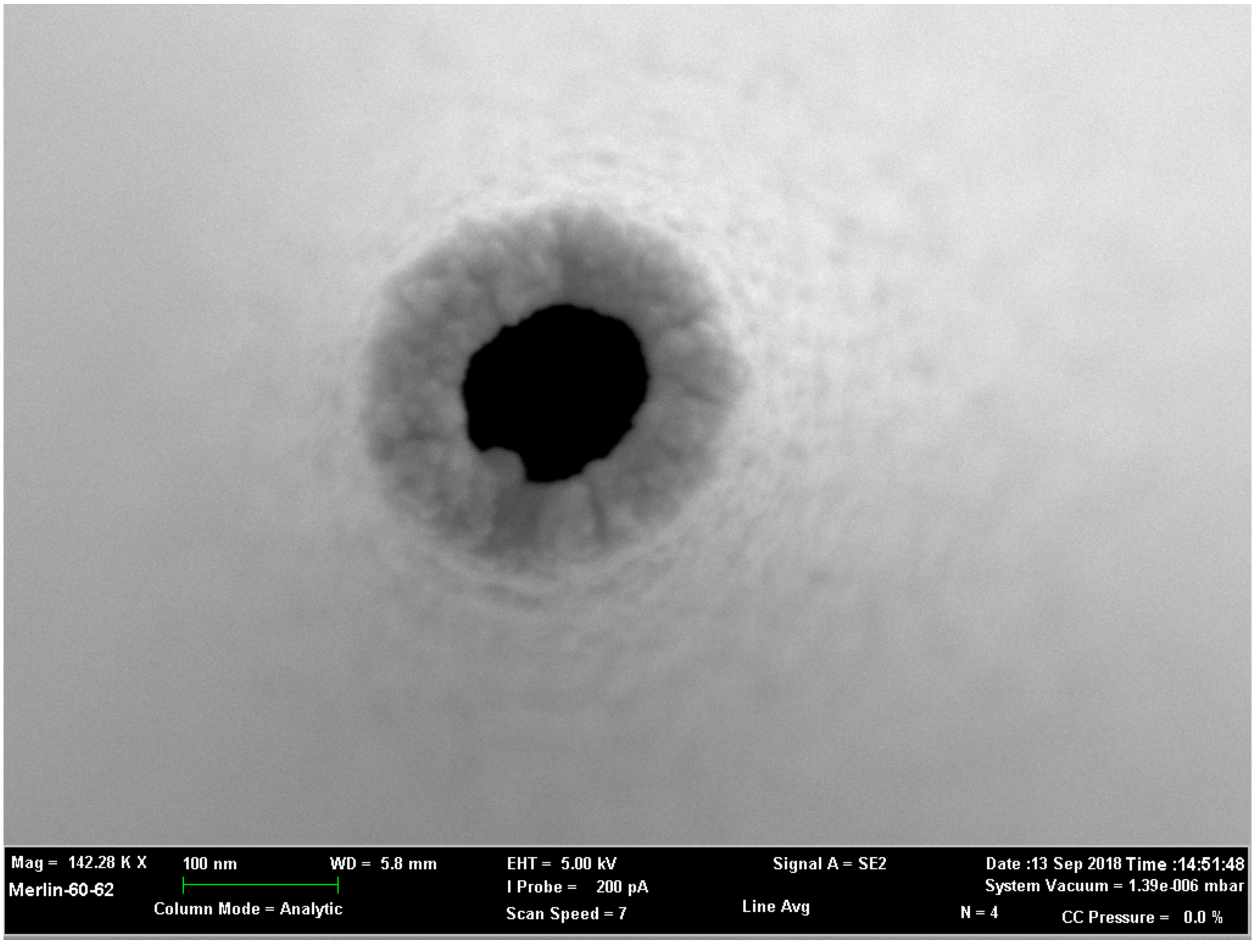

Supplemental Figure 2. Representative scanning electron microscopy micrograph of nanoscale electrospray emitters used in this study. The average diameter was 115 ± 11 nm (n = 9).

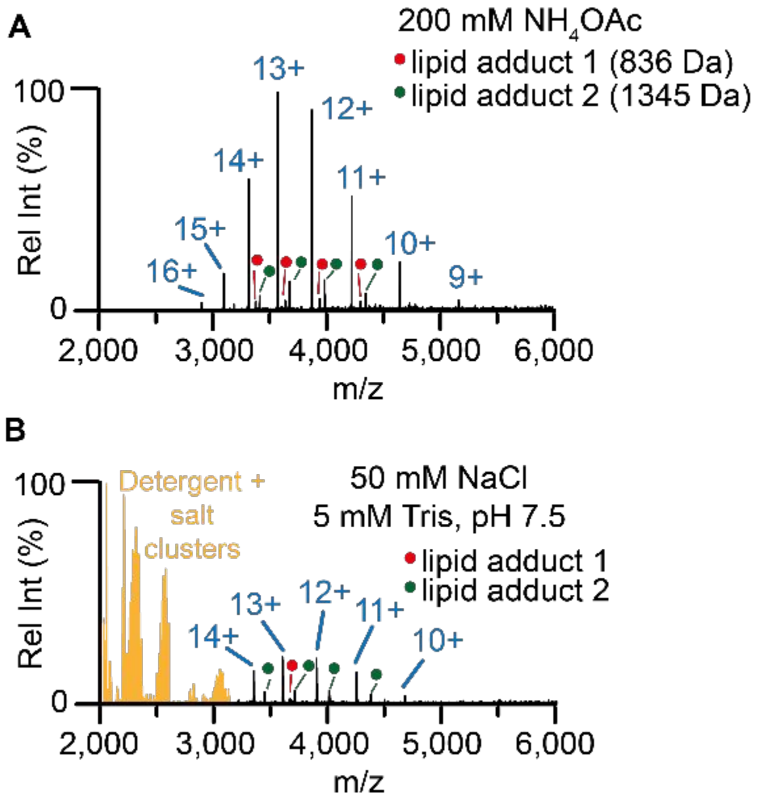

Supplemental Figure 3. Representative native mass spectra of A2aR acquired using approximately 115 nm nanoemitter tips and electrosprayed from (A) 200 mM NH_4OAc, pH 7.5 or (B) 50 mM NaCl, 5 mM Tris, pH 7.5 supplemented with 2x CMC LMNG. Co-purified lipid adducts of 836 and 1345 Da were also observed bound to A2aR.

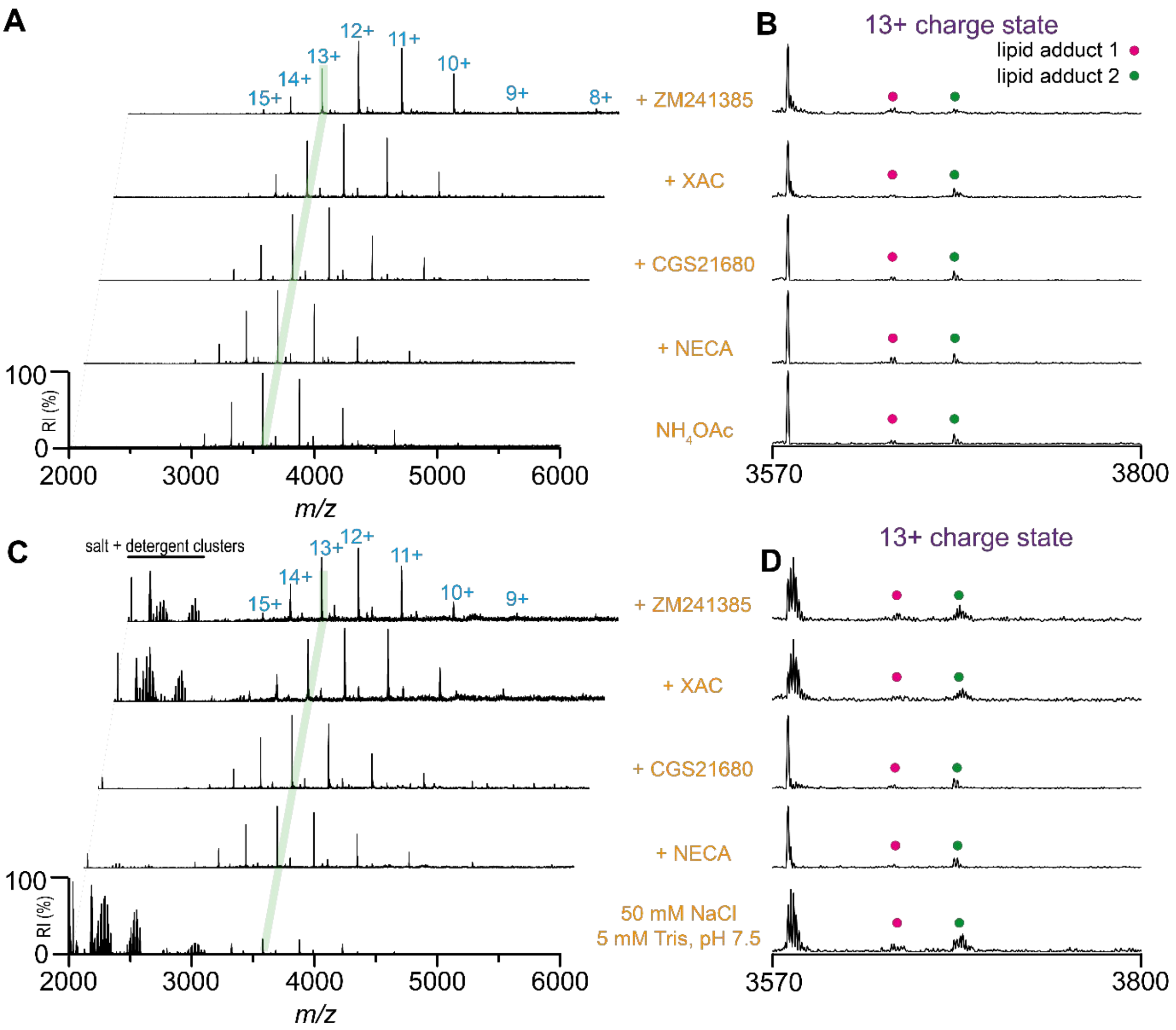

Supplementary Figure 4. Full native MS spectra of A2aR acquired from (A) NH_4OAc or (C) NaCl/Tris solutions supplemented with 2x CMC LMNG in the presence of the ligand indicated. A2aR was incubated with 10 µM ligand for 30 min at 4 ^{o}C prior to data acquisition. Expansion of the *m/z* region around the 13+ charge states are presented for each spectrum on the left from (B) NH_4OAc and (D) NaCl/Tris.

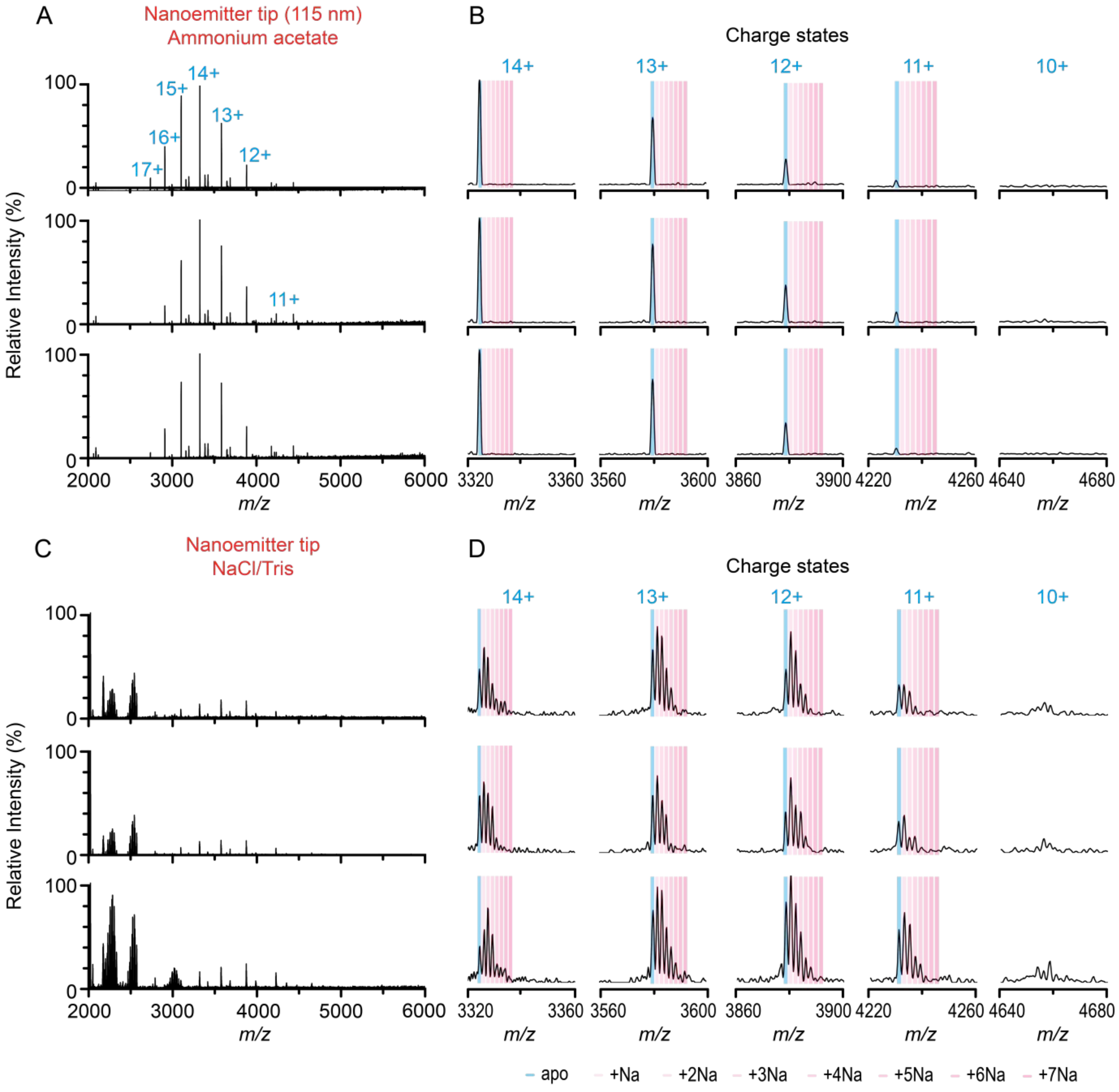

Supplemental Figure 5. Tip to tip nanoemitter variability for A2aR acquired from (A) NH_4OAc or (C) NaCl/Tris electrospray solutions supplemented with 2x CMC LMNG. Expansion of the charge states shows the sodium adduction for (B) NH_4OAc and (D) NaCl/Tris electrospray solutions. No detectable protein ion signal was observed for the 10+ charge state for nanoemitters in NH_4OAc. Spectra were recorded from 3 replicate experiments with three different nanoemitters from the same protein preparation and native MS sample preparation for each experimental condition.

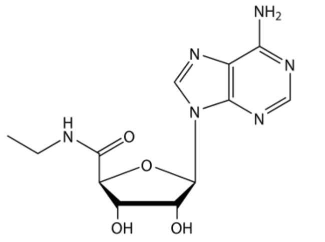

5'-N-Ethylcarboxamidoadenosine

NECA

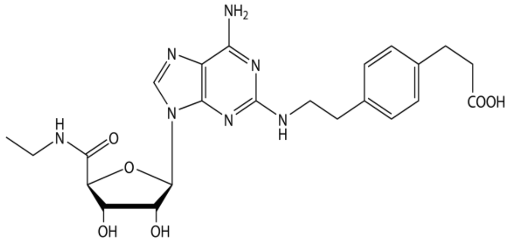

4-[2-[[6-Amino-9-(N-ethyl-β-D-ribofuranuronamidosyl)-9H-purin-2-yl]amino]ethyl]benzenepropanoic acid

CGS 21680

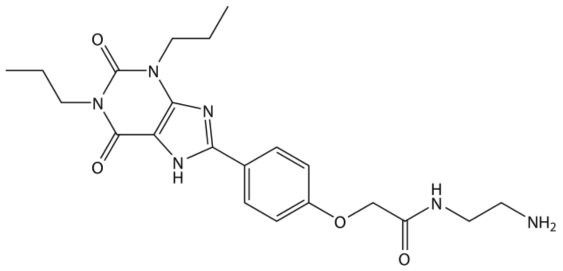

N-(2-Aminoethyl)-2-[4-(2,3,6,7-tetrahydro-2,6-dioxo-1,3-dipropyl-1H-purin-8-yl)phenoxy]-acetamide

XAC

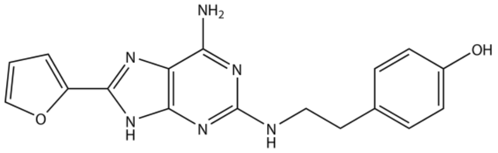

4-(2-[7-Amino-2-(2-furyl)[1,2,4]triazolo[2,3-a][1,3,5]triazin-5-ylamino]ethyl)phenol

ZM 241385

Supplemental Figure 6. Chemical structures of A2aR ligands used in this study: NECA, CGS 21680, XAC, and ZM 241385.

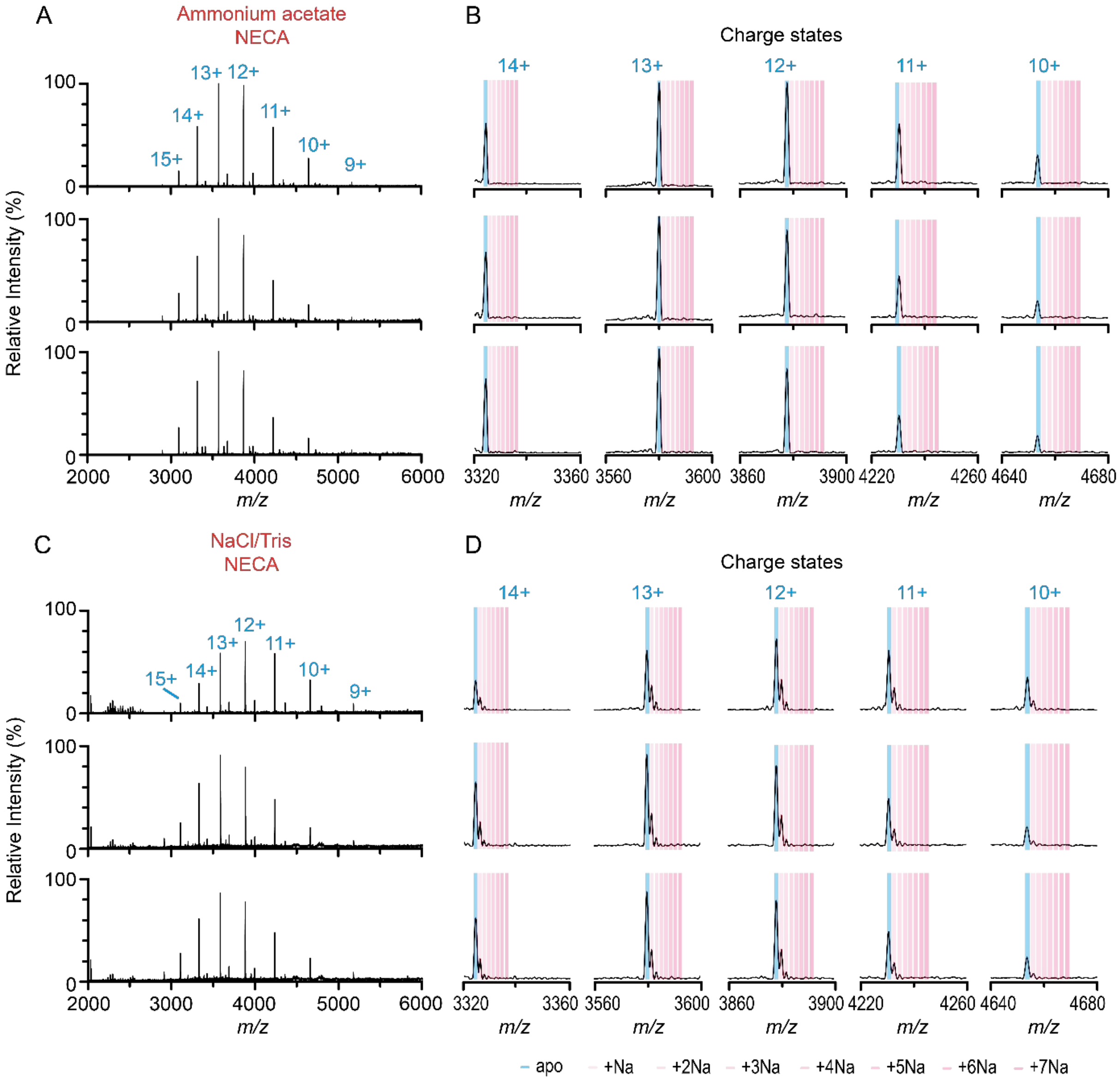

Supplemental Figure 7. Tip to tip nanoemitter variability for A2aR acquired from (A) NH_4OAc or (C) NaCl/Tris electrospray solutions supplemented with 2x CMC LMNG AND 10 µm NECA. Expansion of the charge states shows the sodium adduction for (B) NH_4OAc and (D) NaCl/Tris electrospray solutions. Spectra were recorded from 3 replicate experiments with three different nanoemitters from the same protein preparation and native MS sample preparation for each experimental condition.

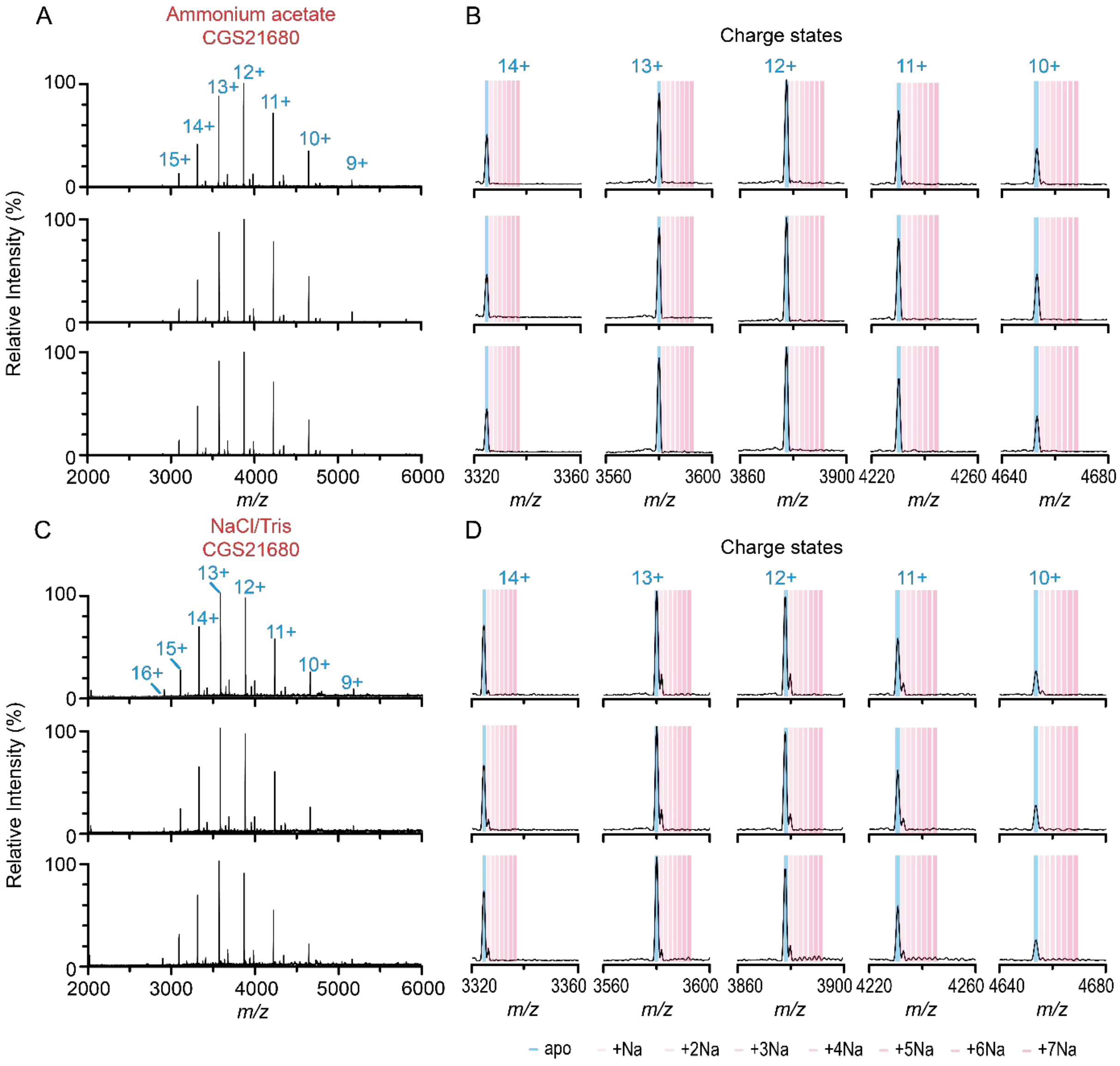

Supplemental Figure 8. Tip to tip nanoemitter variability for A2aR acquired from (A) NH_4OAc or (C) NaCl/Tris electrospray solutions supplemented with 2x CMC LMNG and 10 µM CGS21860. Expansion of the charge states shows the sodium adduction for (B) NH_4OAc and (D) NaCl/Tris electrospray solutions. Spectra were recorded from 3 replicate experiments with three different nanoemitters from the same protein preparation and native MS sample preparation for each experimental condition.

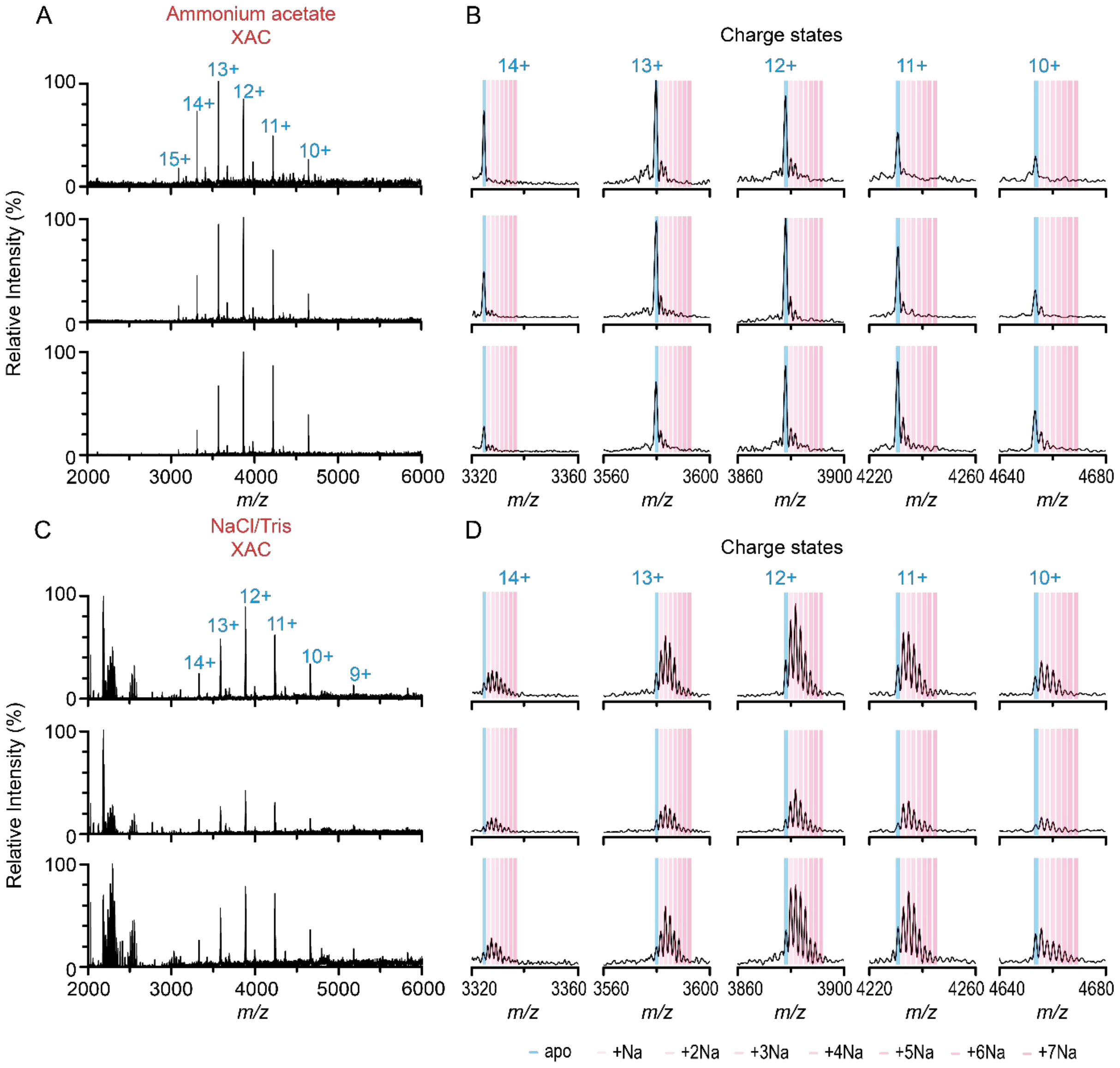

Supplemental Figure 9. Tip to tip variability for A2aR in the presence of 10 µM XAC acquired from (A) NH_4OAc or (C) NaCl/Tris electrospray solutions supplemented with 2x CMC LMNG. Expansion of the *m/z* space for discrete charge states shows the extent of sodium adduction for (B) NH_4OAc and (D) NaCl/Tris electrospray solutions. Spectra were recorded from 3 replicate experiments with three different nanoemitters from the same protein preparation with aliquots taken from the same solution and recorded under the same native MS parameters.

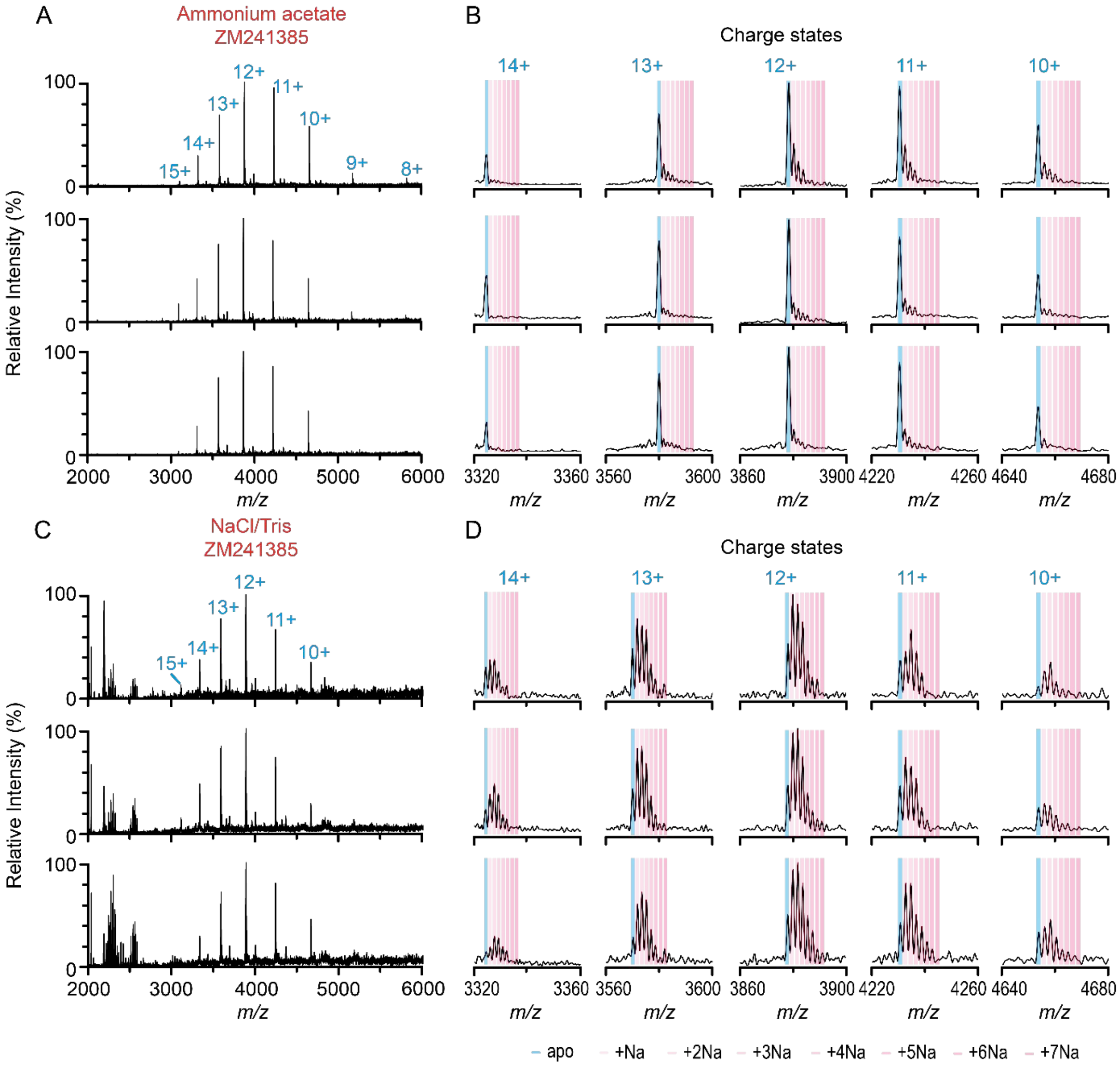

Supplemental Figure 10. Tip to tip variability for A2aR and 10 µM ZM241385 acquired from (A) NH_4OAc or (C) NaCl/Tris electrospray solutions supplemented with 2x CMC LMNG. Expansion of the m/z space for discrete charge states shows the sodium adduction for (B) NH_4OAc and (D) NaCl/Tris electrospray solutions. Spectra were recorded from 3 replicate experiments with three different nanoemitters from the same protein preparation with aliquots taken from the same solution and recorded under the same native MS parameters.

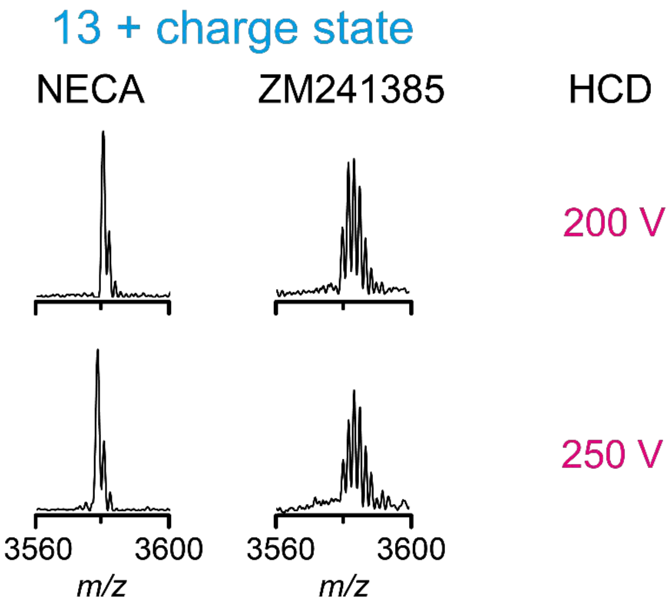

Supplementary Figure 11. Sodium adduct pattern on the 13+ charge state of A2aR in the presence of two different ligands (NECA and ZM241385) illustrates minimal change to the sodium adduction pattern at increased collisional activation energy.

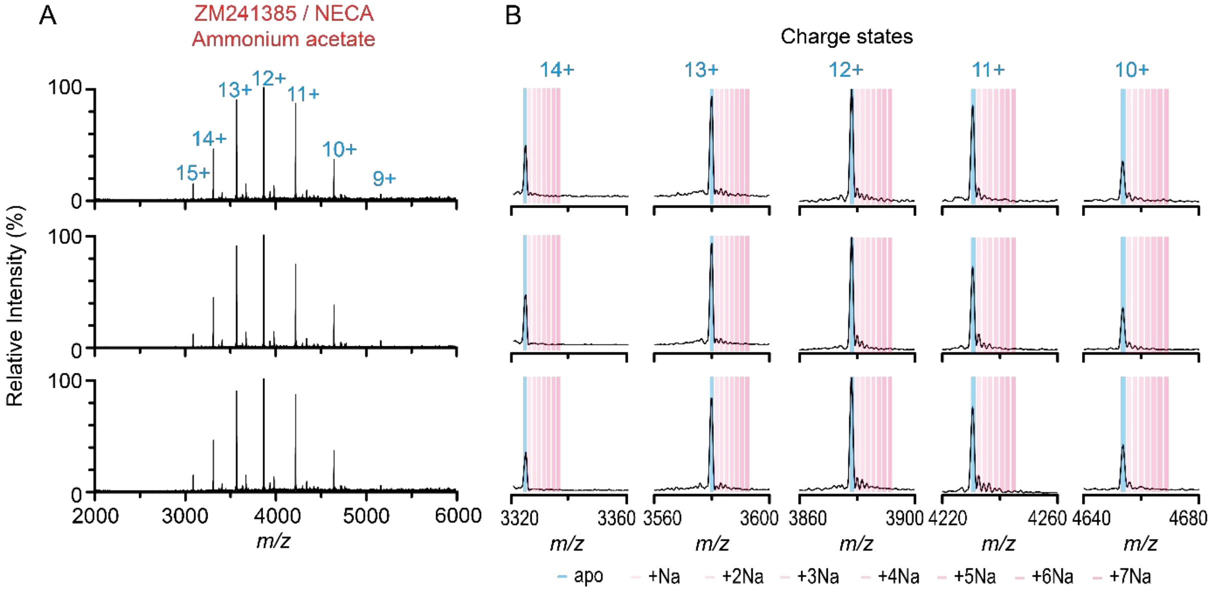

Supplemental Figure 12. Data from ZM241385/NECA competitive inhibition assay in NH_4OAc electrospray buffer showing tip to tip variability. The full mass spectrum is displayed in (A) and the apo state of individual charge states is shown in (B). Reduced sodium binding is apparent compared to incubation with ZM241385 alone. Spectra were recorded from 3 replicate experiments with three different nanoemitters from the same protein preparation with aliquots taken from the same solution and recorded under the same native MS parameters.

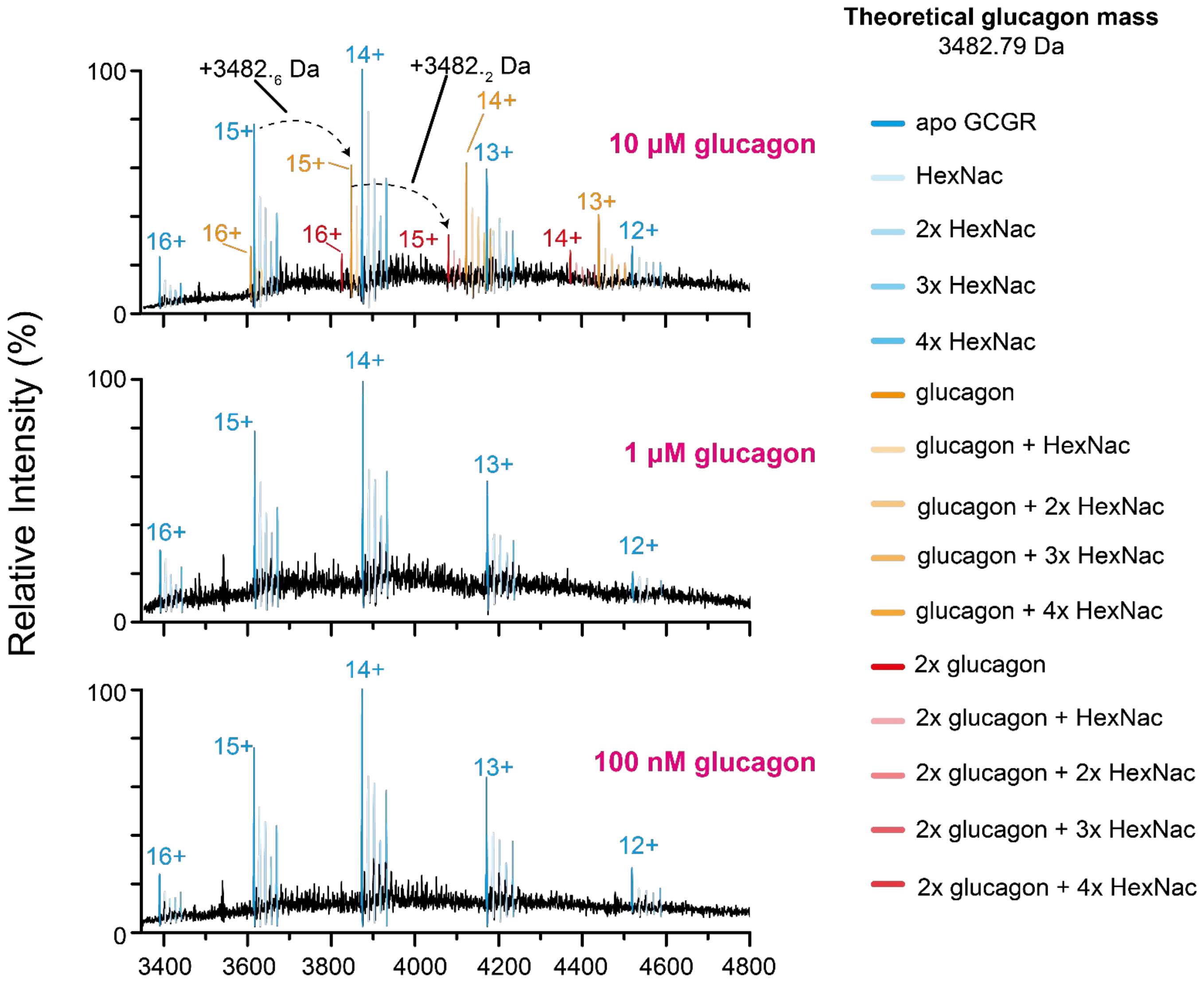

Supplemental Figure 13. Glucagon binding to GCGR demonstrates that the receptor is in an active state. The apo receptor appears as five different glycoforms with zero to up to 4 measurable glycans, highlighted in blue and denoted in the legend. Single glucagon binding to GCGR and its glycoforms is shown (orange) and a low population of two glucagon binding events, likely due to peptide aggregation, is labelled (red).

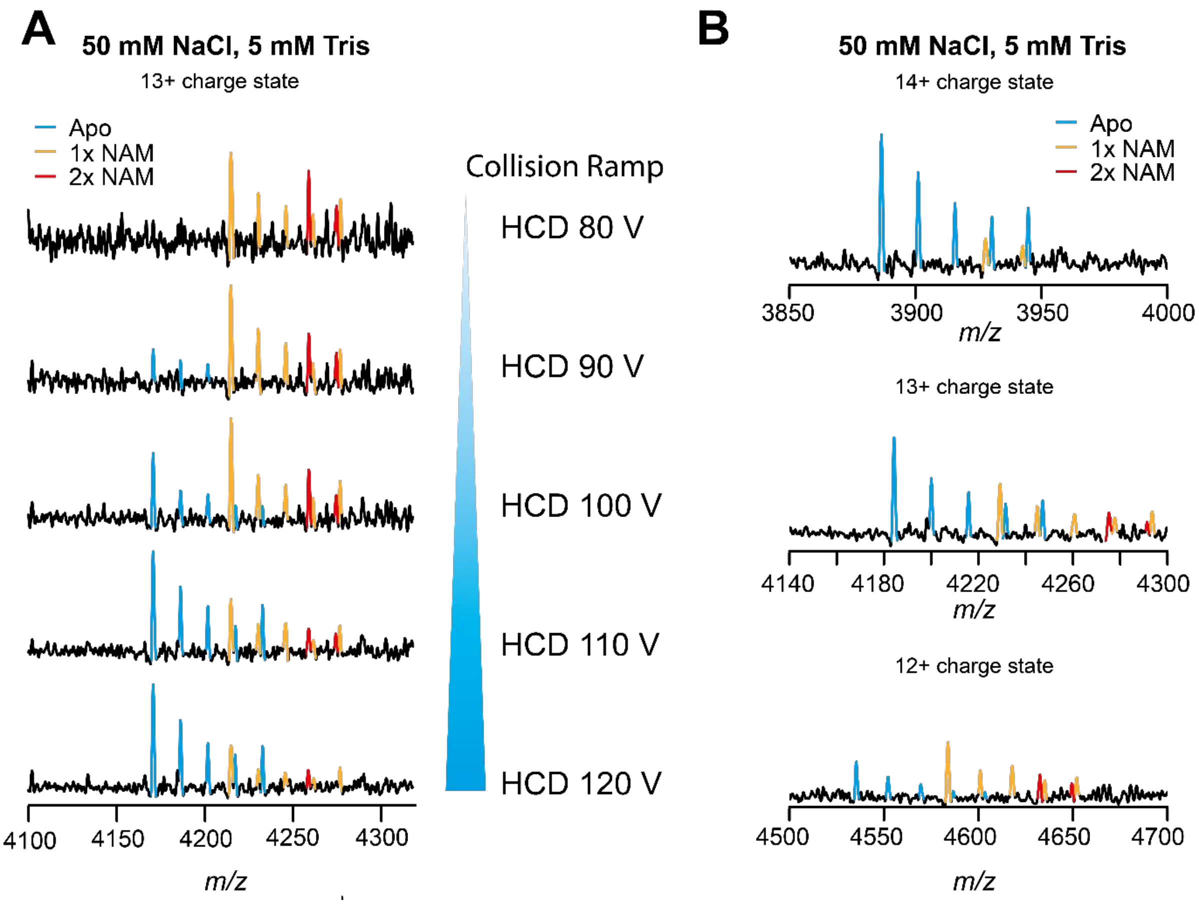

Supplementary Figure 14. Collisional activation energy ramp and charge state dependence of negative allosteric modulator (NAM), NNC0666, binding to GCGR. (A) Collisional ramp from 80 – 120 V in the HCD cell illustrates that the small-molecule NAM readily dissociates from the protein (orange and red peaks reduce as a function of HCD voltage). (B) A comparison of three different charge states at one HCD voltage (110 V) reveals a greater population of NAM binding to GCGR for the lowest charge state (12+) relative to the apo peaks.

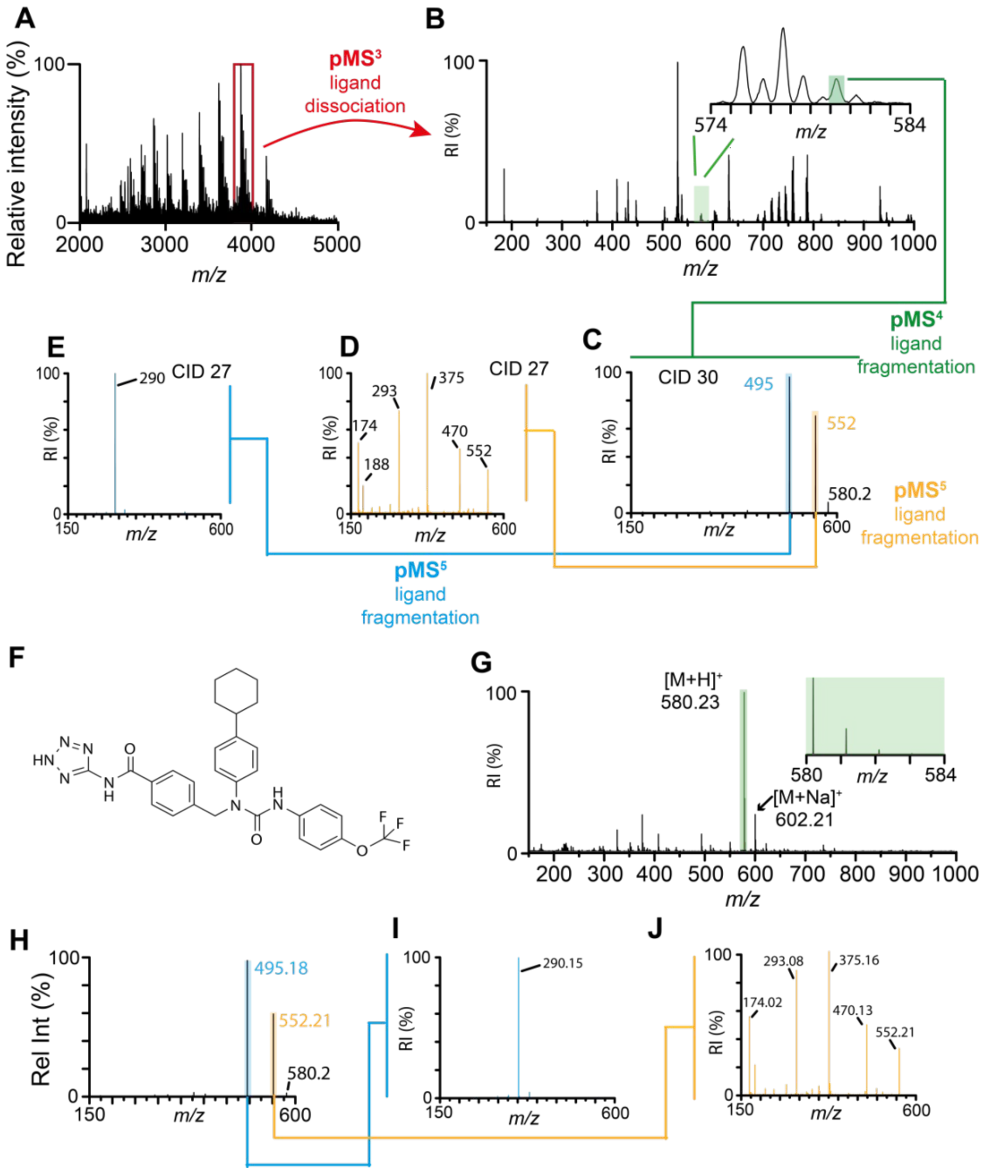

Supplementary Figure 15. **Dissociation and fragmentation of ligands from GCGR using a Thermo Eclipse Orbitrap Tribrid mass spectrometer.** (A) Selection of the 13+ charge state of GCGR and dissociation of ligands yields a range of bound small-molecules (B). Inset in (B) shows species in the *m/z* 574-584 region. (C) Further selection of $[M+H]^+$ = 580 *m/z* (green) and fragmentation in the ion trap yields two major fragments, 552 *m/z* and 495 *m/z*. Subsequent fragmentation of these two products gives rise to spectra (D) and (E). (F) For comparison of the fragmentation patterns, an NNC0666 standard was prepared by dissolving NNC0666 in 50:50 MeOH:H2O and 0.1% TFA and directly infusing this solution into the mass spectrometer. The resulting spectrum (G) was obtained. Inset shows the isotopic distribution of the negative allosteric modulator (NAM), NNC0666. Similar tandem MS experiments (H and I) showed the exact same fragmentation pattern (J) as the ligand dissociated from GCGR (D).

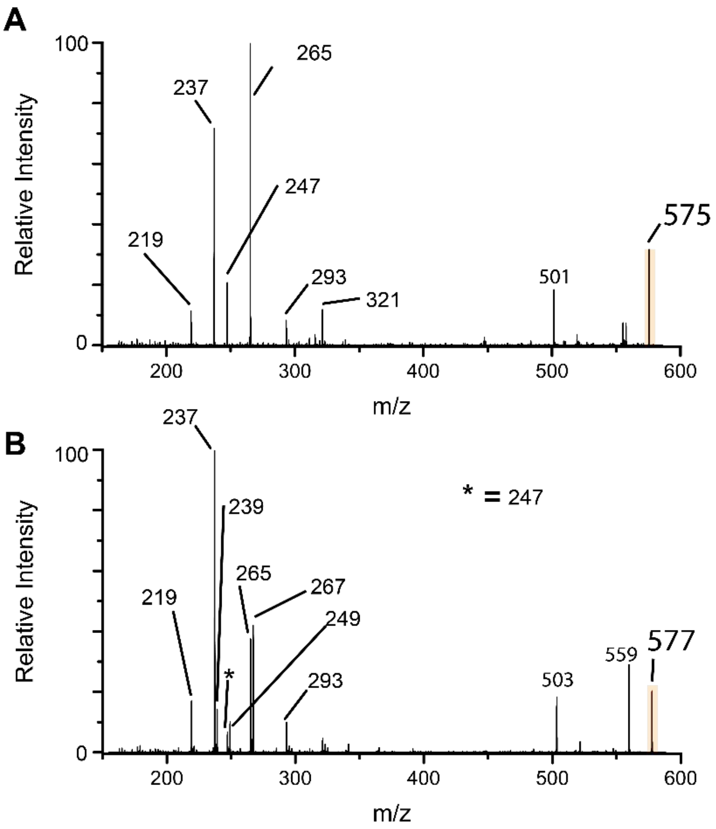

Supplemental Figure 16. Fragmentation spectra of representative species in the m/z 574 -579 range dissociated from GCGR highlighted in the Supplemental Figure 15. The fragments formed from (A) *m/z* 575 and (B) *m/z* 577 are shown.

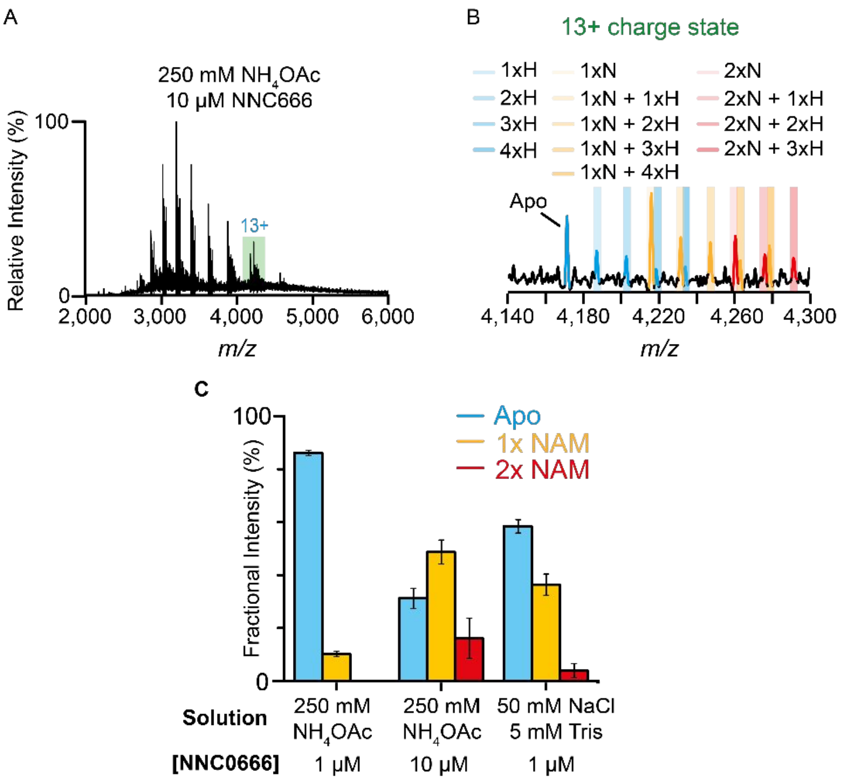

Supplemental Figure 17. Native mass spectrum of GCGR incubated with 10 µM NNC0666 in NH_4OAc. (A) GCGR MS spectrum (*m/z* 2000 – 6000) incubated with 10 µM NNC0666 in 200 mM NH_4OAc. (B) Expansion of the 13+ charge state showing two NNC0666 binding events. (C) Fractional binding of NNC0666 to GCGR under different solution conditions from nanoemitter tips. H = HexNac and N = Negative allosteric modulator (NAM), NNC0666.

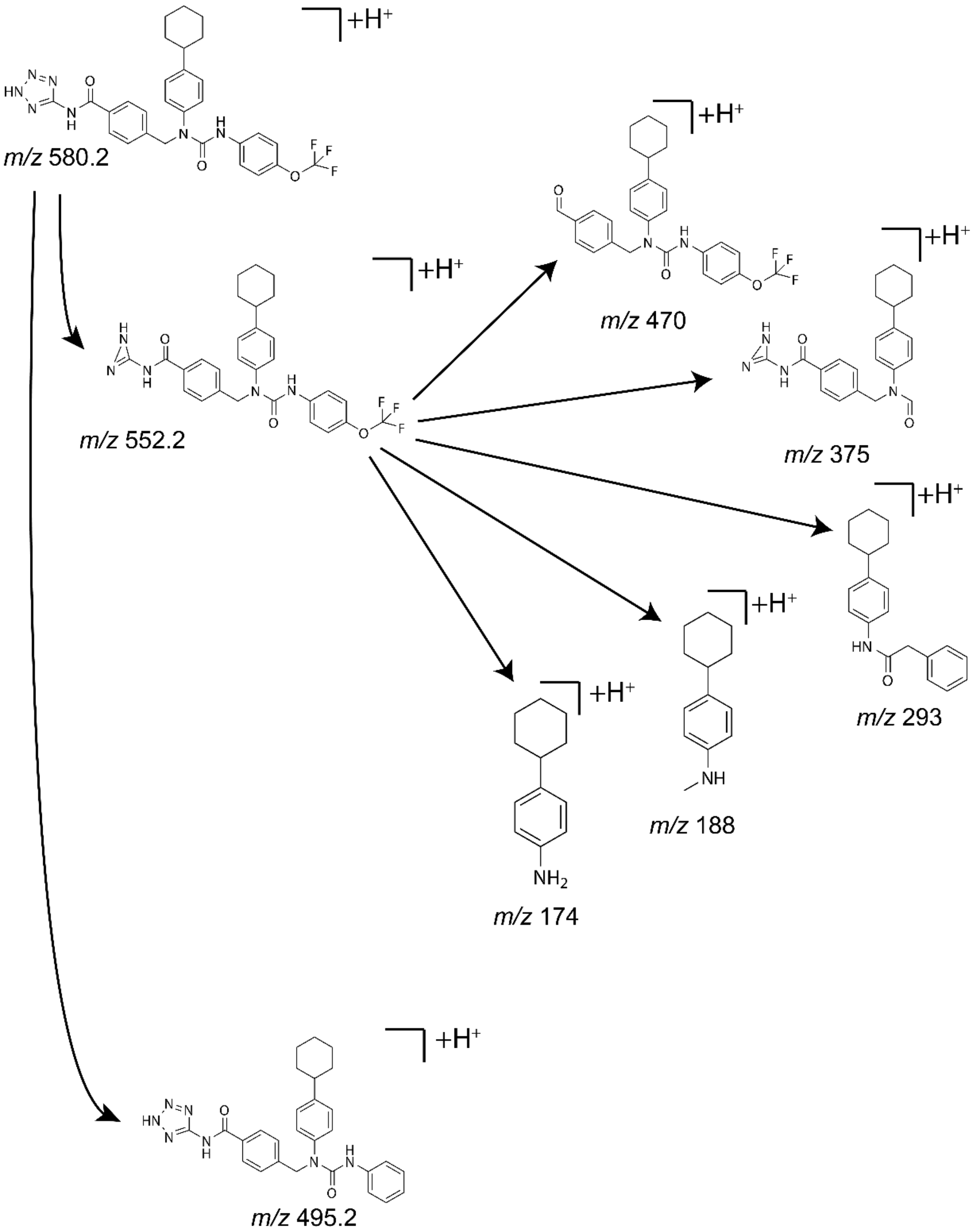

Supplementary Figure 18. Proposed assignment of the ligand fragmentation pathway of NNC0666.

References

1. Nehme, R.; Carpenter, B.; Singhal, A.; Strege, A.; Edwards, P. C.; White, C. F.; Du, H. J.; Grisshammer, R.; Tate, C. G., Mini-G proteins: Novel tools for studying GPCRs in their active conformation. *Plos One* **2017,** *12* (4), e0175642.
2. Urner, L. H.; Liko, I.; Yen, H. Y.; Hoi, K. K.; Bolla, J. R.; Gault, J.; Almeida, F. G.; Schweder, M. P.; Shutin, D.; Ehrmann, S.; Haag, R.; Robinson, C. V.; Pagel, K., Modular detergents tailor the purification and structural analysis of membrane proteins including G-protein coupled receptors. *Nat Commun* **2020,** *11* (1), 564.
3. Susa, A. C.; Lippens, J. L.; Xia, Z. J.; Loo, J. A.; Campuzano, I. D. G.; Williams, E. R., Submicrometer Emitter ESI Tips for Native Mass Spectrometry of Membrane Proteins in Ionic and Nonionic Detergents. *J Am Soc Mass Spectr* **2018,** *29* (1), 203-206.
4. Susa, A. C.; Xia, Z. J.; Williams, E. R., Native Mass Spectrometry from Common Buffers with Salts That Mimic the Extracellular Environment. *Angew Chem Int Edit* **2017,** *56* (27), 7912-7915.
5. Nguyen, G. T. H.; Tran, T. N.; Podgorski, M. N.; Bell, S. G.; Supuran, C. T.; Donald, W. A., Nanoscale Ion Emitters in Native Mass Spectrometry for Measuring Ligand-Protein Binding Affinities. *Acs Central Sci* **2019,** *5* (2), 308-318.
6. Gault, J.; Liko, I.; Landreh, M.; Shutin, D.; Bolla, J. R.; Jefferies, D.; Agasid, M.; Yen, H. Y.; Ladds, M. J. G. W.; Lane, D. P.; Khalid, S.; Mullen, C.; Remes, P. M.; Huguet, R.; McAlister, G.; Goodwin, M.; Viner, R.; Syka, J. E. P.; Robinson, C. V., Combining native and 'omics' mass spectrometry to identify endogenous ligands bound to membrane proteins. *Nat Methods* **2020,** *17* (5), 505-508.
